# Agricultural and empowerment pathways from land ownership to women's nutrition in India

**DOI:** 10.1111/mcn.12995

**Published:** 2020-03-20

**Authors:** Helen Harris‐Fry, Sneha Krishnan, Emma Beaumont, Audrey Prost, Sanghamitra Gouda, Satyanarayan Mohanty, Ronali Pradhan, Suchitra Rath, Shibanand Rath, Shibnath Pradhan, Naba Kishore Mishra, Elizabeth Allen, Suneetha Kadiyala

**Affiliations:** ^1^ Faculty of Epidemiology and Population Health London School of Hygiene & Tropical Medicine London UK; ^2^ Institute for Global Health University College London London UK; ^3^ Development Corner Consulting Pvt. Ltd. Bhubaneswar India; ^4^ Digital Green Bhubaneshwar India; ^5^ Ekjut Chakradharpur India; ^6^ Voluntary Association for Rural Reconstruction and Appropriate Technology Keonjhar India

**Keywords:** agriculture, diet, land size, mediation, undernutrition, women's empowerment

## Abstract

Land size is an important equity concern for the design of ‘nutrition‐sensitive’ agricultural interventions. We unpack some of the pathways between land and nutrition using a cross‐sectional baseline survey data set of 4,480 women from 148 clusters from the ‘Upscaling Participatory Action and Videos for Agriculture and Nutrition’ trial in Keonjhar district in Odisha, India. Variables used are household ln‐land size owned (exposure) and maternal dietary diversity score out of 10 food groups and body mass index (BMI; kg/m^2^) (outcomes); and mediators investigated are production diversity score, value of agricultural production, and indicators for women's empowerment (decision‐making in agriculture, group participation, work‐free time and land ownership). We assessed mediation using a non‐parametric potential outcomes framework method. Land size positively affects maternal dietary diversity scores [*β* 0.047; 95% confidence interval (CI) (0.011, 0.082)] but not BMI. Production diversity, but not value of production, accounts for 17.6% of total effect mediated. We observe suppression of the effect of land size on BMI, with no evidence of a direct effect for either of the agricultural mediators but indirect effects of *β* −0.031 [95% CI (−0.048, −0.017)] through production diversity and *β* −0.047 [95% CI (−0.075, −0.021)] through value of production. An increase in land size positively affects women's decision‐making, which in turn negatively affects maternal BMI. The positive effect of work‐free time on maternal BMI is suppressed by the negative effect of household land size on work‐free time. Agriculture interventions must consider land quality, women's decision‐making and implications for women's workload in their design.

**Key messages**

- Our results indicate a weak household land size to maternal nutrition gradient.
- Although land could improve some agriculture and women's empowerment indicators, these may act as suppressors of maternal nutritional outcomes, especially BMI.
- Agriculture programmes aiming to increase household productive assets, such as land transfer programmes, must be designed to consider quality of the transferred land and access to agricultural inputs and their implications for women's and intra‐household allocation of labour.

## 1 INTRODUCTION

There is increasing evidence that agricultural interventions can be designed to improve diets in undernourished, low‐income communities (Ruel, Quisumbing, & Balagamwala, 2018). Some of these ‘nutrition‐sensitive’ agricultural interventions have improved dietary quality through crop biofortification (De Brauw et al., 2018) or diversifying crop and livestock production (Darrouzet‐Nardi et al., 2016; Olney et al., 2016; Schreinemachers, Patalagsa, & Uddin, 2016), whereas others have used value‐chain approaches and agricultural technology to increase household incomes (Alaofè, Burney, Naylor, & Taren, 2016; Le Port et al., 2017). Some interventions, such as sustainable intensification (Pretty & Hine, 2001), rely on participants' ownership of sizeable landholdings, whereas others, such as small livestock interventions and kitchen gardens (Darrouzet‐Nardi et al., 2016; Olney et al., 2016; Schreinemachers et al., 2016), require smaller parcels of land. A common theme is that most, if not all, agricultural interventions rely on households to have access to some cultivable land.

Consequently, land size is an important equity concern for the design of agricultural interventions. Some evidence indicates that nutrition‐sensitive agriculture interventions improve health outcomes to a greater extent in better‐off groups (Jones & de Brauw, 2015; Le Port et al., 2017), suggesting that interventions need to be intentionally designed to be pro‐poor. Historically, in many agrarian societies across the world, wealthier landowners have employed poorer, lower class or caste, landless groups to work on their land in a patron–client relationship that entrenched socio‐economic inequalities (Cameron, 1995; Lawry, 1990; Scott, 1972). A wealth gradient in nutritional status is commonly observed in national surveys, and determinants analyses have commonly identified wealth as the strongest predictor of dietary quality (Aemro, Mesele, Birhanu, & Atenafu, 2013; Harris‐Fry et al., 2015), indicating that these poorer, landless groups have poorer nutritional status (Arimond & Ruel, 2004). Taken together, this suggests that nutrition‐sensitive agriculture interventions may be both less relevant but also more needed in poor households with little or no land.

However, the importance of land size for supplying households with adequate nutrition remains unclear. A recent synthesis of evidence found mixed results on the associations between land ownership and dietary intakes (Shankar, Poole, & Bird, 2019), including small positive (Mulmi et al., 2017; Viswanathan, David, Vepa, & Bhavani, 2015), null (Bhagowalia, Kadiyala, & Headey, 2012; Harris‐Fry, 2017; Harris‐Fry et al., 2015) and even small, negative (Hossain, Jimi, & Islam, 2016) associations.

This heterogeneity may be because the linkages from agricultural production to consumption are highly mixed (Ruel et al., 2018). Differences in market access and food storage facilities may explain this, with limited market access cornering households into consuming their own production and strengthening the production–consumption pathway in some places more than others (Hoddinott, Headey, & Dereje, 2015). On the other hand, estimates of effects of land on diets may be confounded by wealth, particularly because current evidence is based on observational data so has been unable to isolate causal effects (Shankar et al., 2019). It is likely that wealthier households can afford to cultivate or buy more land and can also afford more adequate diets. Moreover, the land tenure landscape is changing in many places, with land redistribution and titling programmes, migration to urban areas and increasing reliance on non‐farm work in rural areas (Holden & Otsuka, 2014; Rigg, 2006). These processes could be weakening the links between land ownership and diets.

Differential effects of land on diets may also be explained by varied roles of women in agriculture. Women have historically had less access to land than men, particularly in patrilineal contexts where sons traditionally inherit land (Doss, Kovarik, Peterman, Quisumbing, & van den Bold, 2015). Perhaps as a result of this, many women also have less control over the use of land across the value chain: from decision‐making over agricultural processes, processing, marketing and sale and use of agricultural outputs (Meinzen‐Dick et al., 2012). Wide variance in these gender roles across contexts, over seasons and at each point in the value chain (Meinzen‐Dick et al., 2012) could explain differences in the effects of land on nutritional intakes and outcomes.

With newer nutrition‐sensitive agricultural approaches being tried and tested, understanding the pathways from land ownership to women's nutrition is important to inform the design of equitable programmes. In our study, we unpack some of the hypothesised connections between land and nutrition using a detailed data set from rural Odisha, in Eastern India. Specifically, we contribute to the literature in the following ways: first, we examine if owning land, a critical input into agriculture, affects maternal nutritional outcomes [dietary diversity and body mass index (BMI)] in a rural Indian context, where agriculture is a main source of livelihoods. By considering land as an asset, we look at total land ownership, rather than land cultivated as our exposure variable. Second, recognising the heterogeneity in effects of land ownership on nutrition outcomes reported in other studies, we unpack the relationship between household land ownership and maternal nutritional outcomes. Specifically, we explore if indicators of agricultural production and women's empowerment mediate this relationship. We further make a novel contribution to this field by applying a robust causal inference framework to our mediation analysis.

## 2 METHODS

We use a detailed cross‐sectional data set from rural Odisha, India, containing information on landholdings, maternal and nutritional status, agricultural production and women's empowerment. Data belong to the baseline survey of the ‘Upscaling Participatory Action and Videos for Agriculture and Nutrition’ (UPAVAN) trial. Full details on data collection procedures are given in the UPAVAN trial protocol (Kadiyala et al., 2018). We follow ‘STROBE‐NUT’ (STrengthening the Reporting of OBservational studies in Epidemiology—Nutritional Epidemiology) guidelines (Lachat et al., 2016).

### 2.1 Study setting and population

The survey was conducted between November 2016 and January 2017 in 148 clusters (villages and surrounding hamlets) from four administrative blocks: Patna, Ghatgaon, Keonjhar Sadar and Harichandanpur, in the Keonjhar district of Odisha state, India. Rural livelihoods predominate, agriculture is the main source of income (Odisha District Website, 2019), and there is a high burden of undernutrition. Around a third of women in Keonjhar are underweight (BMI <18.5 kg/m^2^), and 40% are anaemic (haemoglobin <12.0 g/dl; NFHS‐4, 2016).

We sampled households containing at least one child aged 0–23 completed months and interviewed the female primary caregivers (aged 15–49 years) and their husbands (or other adult males, if the husband was not available). We aimed to sample 32 households per cluster, giving an intended sample size of 4736 mother–child dyads. The sample size was calculated to detect differences in the two primary outcomes of the trial (percentage of children aged 6–23 months consuming at least four out of seven food groups per day and maternal mean BMI). We excluded any households where the mother or child had a discernible disability affecting their anthropometric measurements or ability to respond to the questionnaires. Data on household food expenditures were collected on a randomly selected 50% of the sample, using a Household Expenditure and Consumption Survey.

Informed consent was obtained from all respondents.

### 2.2 Data collection

Nine teams of local, trained interviewers interviewed the respondents using a pretested questionnaire translated into Odia language. We trained interviewers for 3 weeks before the start of data collection. Interviewers measured women's height using Seca 213 Stadiometers, weight using PLAX‐Cruzer scales, and mid–upper arm circumference (MUAC) using Seca circumference tapes. We standardised anthropometric measurements by comparing against a ‘gold standard’ measurer and calculating inter‐ and intra‐technical error of measurement. We also standardised dietary diversity assessments, by asking each interviewer and a gold standard measurer to question the same woman using a set probing technique. Weaker interviewers were given additional training.

We collected data on paper questionnaires, and a quality assurance team checked them for plausibility and logic at the field site before double entry into a database in the nearest city (Bhubaneswar). The data management team observed 10% of the interviews to ensure data quality and adherence to procedures, and took repeat measurements for a subset of questions in 20% of households.

The variables used in this study are described below and in more detail in the Supporting Information:

- **Exposure:** Natural‐log (ln) household land size owned, as reported by the respondent. Land includes homestead land, agriculture land and any other land. Households may have a record of rights, a share of ancestral land or land with no record, including encroached land. ‘Ownership’ is based on the respondents' perceptions of whether they own the land and does not distinguish between whether or not they have a bundle of rights [access, withdrawal, management, exclusions and alienation (Quisumbing et al., 2015)] to the land.
- **Nutritional outcomes:**
– Maternal dietary diversity score as a count out of 10 food groups consumed by female caregivers aged 15–49 years, calculated using the Minimum Dietary Diversity for Women (MDD‐W; FAO, 2014)
‐BMI (kg/m^2^) of non‐pregnant, non‐post‐partum female caregivers aged 15–49 years
- **Hypothesised agricultural and women's empowerment mediators:**
‐Value of agriculture production: ln‐value of total agricultural production in the last three agricultural seasons (in the last 12 months), in 1,000 Indian Rupees. Production from all cultivated land (owned, rented, shared or other arrangements such cultivating on an extend family member's land or community land) included.
‐Agriculture production diversity: Count of 10 food groups produced, regardless of land ownership status, in the last three agricultural seasons (in the last 12 months) by households in any quantity.
‐Women's decision‐making: Women's self‐reported involvement (to at least some extent of involvement) in ≥2 versus <2 productive decisions in the household, out of four possible decisions (Malapit et al., 2015)
‐Women's group participation: Women's self‐reported active participation in any community groups (Malapit, Kovarik, et al., 2015)
‐Women's time use: Amount of work‐free time that women have (<10.5 vs. ≥10.5 h of work) based on a 24‐h time‐use recall (Malapit, Kovarik, et al., 2015)
‐Women's land ownership: Women's self‐reported land ownership, in two categories: none versus any (joint or sole) ownership.

In addition to the variables of interest on the pathway from land to maternal nutrition, we also used the following variables that we identified well known a priori as confounders: caste group (Coelho & Belden, 2016), years of maternal education (Subramanian & Smith, 2006), count of household assets (Subramanian & Smith, 2006), household size (Rashid, Smith, & Rahman, 2011), female‐only households (Rashid et al., 2011) and maternal age ( Harris‐Fry et al., 2015). The asset score includes the following 15 assets: high cost consumer durables, low cost consumer durables, jewellery, mobile phone, electricity, computer, internet, motorbike, mechanised agricultural assets, business assets, high‐quality fuel type, finished flooring, finished roofing, finished walls and toilet. This score excludes assets that are highly correlated with land ownership: house, bicycle, small livestock, large livestock and non‐mechanised equipment.

### 2.3 Data analysis

All analyses were conducted in Stata SE 15. Descriptive statistics are presented using means [standard deviations (SDs)], median [inter quartile range (IQR)] or percentages, for normally distributed continuous, non‐normally distributed continuous and binary variables, respectively. We visualise patterns of dietary diversity and BMI with increasing land size using local polynomial smoothing estimates with 95% confidence intervals (CIs).

Given that we have a number of potential binary mediators and that there are recognised limitations to structural equation modelling in identifying mediation in nonlinear models (Imai, Keele & Tingley, 2010), we assess mediation using the ‘potential outcomes framework’. The potential outcomes framework is a non‐parametric approach that applies the logic of counterfactuals to identify mediated effects (Little & Rubin 2000; Mithas & Krishnan 2009; Rubin 2011).

The aim of mediation analysis is to quantify how much of the effect of an exposure acts through a particular pathway. To further explain the approach used here, let *M*(*x*) denote the potential value of the mediator under the exposure status *x*, *Y*(*x*, *m*) represent the potential outcome of *Y* when *X* = *x* and *M* = *m* and *Y*(*x*, *M*(*x′*)) indicate the counterfactual value of *Y* that would be observed if *X* was set to *x* and *M* was set to its potential outcome that would be observed if *X* was set to *x'.*

The quantity of interest (the indirect effect) can be defined for an individual for any two levels of exposure as *Y*(*x*, *M*(*x*_1_)) − *Y*(*x*, *M*(*x*_0_)), that is, the change that would occur to the outcome if one changed the mediator from the value that would be realised under one condition, *M*(*x*_0_), to the value that would be observed under another condition, *M*(*x*_1_), while holding the exposure status at *x.*

As an example, consider maternal dietary diversity as the outcome, decision‐making as the binary mediator, and land ownership as the exposure. Here we are interested in the difference between the woman's dietary diversity score for a fixed value of land ownership for the two possible values of decision‐making. Although we can observe the dietary diversity score for a woman with a given value of land ownership and observed decision‐making score, we cannot observe what the woman's dietary diversity score would be if the decision‐making score had been different (with the same land ownership)—the counterfactual.

Although only one combination can be observed for each woman and all other possible combinations are counterfactual for that woman, the combinations that are counterfactual for one woman are observed for other women. Therefore, fitting models allows us to predict the unobserved values (counterfactuals) for any woman given her characteristics and characteristics of similar women in the sample. Here we use non‐parametric simulations to estimate these counterfactuals and their uncertainty.

The simulation process follows a four‐step algorithm designed by Imai et al. (2010) and is as follows: in the first step, models are fitted to estimate the effect of the exposure on the outcome and mediator variables. In Steps 2 and 3, model parameters are simulated from their sampling distribution to determine the potential (unobserved) values of the mediator and the resulting average causal mediated effect (indirect effect), the average direct effect and the average total effect of the intervention on the outcome of interest. In the fourth step, summary statistics and CIs are calculated.

In order to make inferences on any mediated effects, two ‘assumptions of sequential ignorability’ must be satisfied (Imai, Keele, & Yamamoto, 2010). Broadly speaking, the first assumption relates to the ignorability of the exposure: potential mediators and outcomes must not affect exposure. The second assumption is that the mediator is ignorable given the observed exposure and covariates. The first assumption is satisfied if the exposure precedes the mediator and outcomes in time and is satisfied here. In our sample, 90.9% of the sample owned land in ancestral name. Only 4.9% reported owning land with a record of rights, and 1.3% reported having owned a piece of land without a record. Because land size owned is predominantly determined by inheritance and government allocations (which mostly occurred between 1960 and 2013), in this context, rather than women's empowerment and agriculture productivity, we assume that this exposure precedes the hypothesised mediators, at least in the short run. As such, given the counterfactual framework and the ignorability of the first assumption, following the methodology, we use the term effect rather than association while presenting our results. The second assumption requires that there is no unmeasured confounding anywhere along the causal pathway (Imai, Keele & Yamamoto, 2010). However, as there is no way to rule out the presence of confounding, statistical sensitivity analyses are required that can provide an indication of whether results may be violating this assumption (Keele, 2015). Given this, as is the case with most non‐randomised counterfactual based analyses, our analysis does not account for time‐variant unobservables, and therefore, estimates are not completely free from bias.

Analysis was carried out using the Stata ‘mediation’ package designed specifically for mediation analysis based on the potential outcomes framework (Hicks & Tingley, 2011). Within the package, the ‘medeff’ command was used to implement the algorithm described above. A second command, ‘medsens’, runs the sensitivity analysis to investigate if the results were subject to violations of the assumptions of sequential ignorability (Hicks & Tingley, 2011). This was run if there was evidence of mediation in the initial analysis.

### 2.4 Ethical considerations

Ethical approvals were obtained from Odisha government's Institutional Review Board, Research and Ethics Committee, Department of Health and Family Welfare, Government of Odisha (date approved 3 September 2016, Letter No. 141/SHRMU) and the LSHTM Interventions Research Ethics Committee (date approved 10 October 2016, Reference No. 11 357). The trial is registered with ISRCTN (doi: https://doi.org/10.1186/ISRCTN65922679).

## 3 RESULTS

### 3.1 Respondent characteristics

We visited 5,427 households and interviewed 4,480 households, giving an 83% response rate. Two hundred eighty‐two women were pregnant or post‐partum (gave birth <42 days before the interview), so they were excluded from analyses with BMI as an outcome.

Descriptive statistics on the exposures, outcomes, confounders, mediators and other sociodemographic characteristics are given in Table 1.

**TABLE 1:** Respondent characteristics

Characteristic	Statistic	*n*
**Household land ownership**		
Owns any land, %		
No	5.8%	259
Yes	94.0%	4,210
Acres of land owned (if any), median (IQR)	1.15 (0.62 to 2.05)	4,193
Does not own land, %	5.8%	259
< 2.5 acres	74.9%	3,354
2.5–5 acres	14.7%	657
>5 acres	4.1%	182
**Diets**		
Women's dietary diversity score out of 10 groups, mean (SD)	3.7 (1.9)	4,471
‘Adequate’ dietary diversity; ≥5 out of 10 food groups, %	21.3%	956
Maternal body mass index (BMI), mean (SD)	19.2 (2.5)	4,478
Daily household food expenditures in Indian rupees, median (IQR)	108 (73 to 159)	2,217
**Agricultural production**		
Production diversity out of 10 food groups in the last 3 agricultural seasons, mean (SD)	3.6 (1.4)	4,472
Value of agricultural production in the last 3 agricultural seasons in 1000 Indian rupees, median (IQR)	4.5 (2.1 to 8.7)	4,473
**Women's empowerment**		
Women have input into some or all of the decision, %		
Food crop farming	67.4%	3,018
Cash crop farming	18.0%	808
Livestock raising	68.0%	3,045
Non‐farm economic work	29.4%	1,318
Women have at least some input in two or more decisions, %	63.4%	2,838
Women active member in at least one community group, %	30.0%	1,339
Women worked less than 10.5 h in last 24 h, %	40.2%	1,800
Women own land, %		
None owned	83.1%	3,548
Jointly owned	15.8%	676
Solely owned	1.05%	45
**Socio‐** **economic** **status**		
Maternal education in years, mean (SD)	6.4 (4.5)	4,477
Caste group, %		
Scheduled caste	9.1%	406
Scheduled tribe	58.4%	2,614
Other backward class	30.0%	1,346
Other	2.4%	106
Asset score based on 15 assets, mean (SD)	5.6 (2.7)	4,350
**Other demographic indicators**		
Mother's age in completed years, mean (SD)	24.5 (4.0)	4,467
Number of household members, mean (SD)	5.4 (2.1)	4,477
Female‐only household, %	4.0%	178

Abbreviations: IQR, inter quartile ratio; SD, standard deviation.

Few households (6%) had no land. Around three quarters own a small plot <2.5 acres, but only 17% of women reported owning any land themselves. Maternal diets were inadequate, with around four fifths not consuming the recommended five or more food groups per day. Diversity of diets and agricultural production were similar (mean: 3.7 and 3.6 food groups, respectively), and the value of agricultural production was also strikingly low (median: 4,469 INR/year). Most women were involved in household decisions on agricultural activities, but their work burdens were high; less than half (40%) worked less than 10.5 h in a day.

### 3.2 Associations between land size and maternal nutrition

**FIGURE 1:**
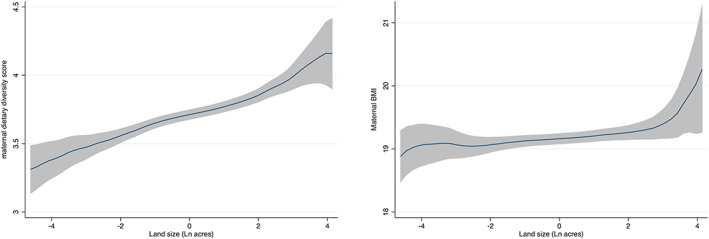
Maternal dietary diversity scores and maternal body mass index by size of landholding. Grey shaded areas are 95% confidence intervals. Maternal dietary diversity *n* = 4,421; body mass index (BMI) *n* = 4,145

Figure 1 shows that women from households with the largest landholdings consumed around one more food group per day, compared with households with the smallest landholdings, but no evidence of a gradient with land size for BMI apart from perhaps women with the largest landholdings (Figure 1).

### 3.3 Pathways from land to maternal dietary diversity through agriculture and women's empowerment

**FIGURE 2:**
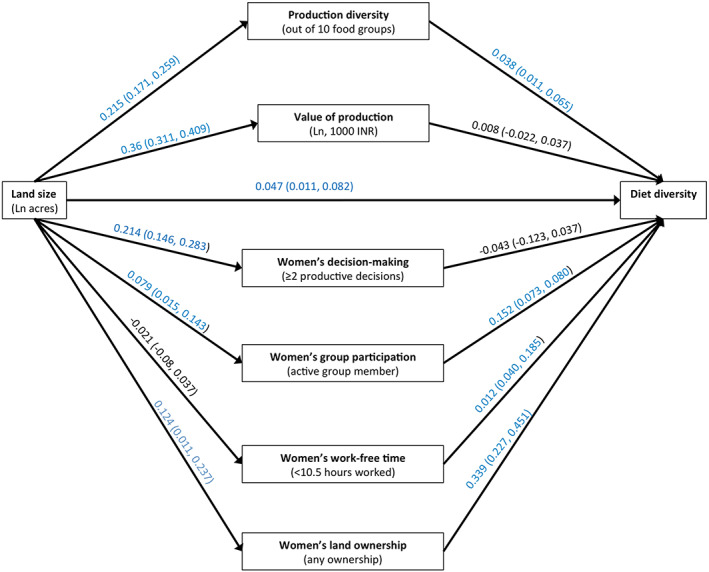
Pathways from land size to maternal diet diversity: Estimations from potential outcomes framework analysis. All coefficients are from linear or logit regression regressions. All models adjust for caste group, years of maternal education, asset score, household size, female‐only households, maternal age and clustered study design. Estimations of associations between mediators and outcome are adjusted for exposure (ln land size)

Figure 2 gives the total effect of (ln) land size on maternal dietary diversity scores along with the effects of land size on the hypothesised mediators and the effects of the mediators on maternal dietary diversity (adjusted for land size). Consistent with Figure 1, there is evidence of positive total effect of land size on maternal dietary diversity scores [*β* 0.047; 95% CI (0.011, 0.082)]. Land size affects all mediators except women's work‐free time.

Table 2 gives the estimates of the direct effect of land size on maternal dietary diversity, the indirect effect through each hypothesised mediator, and the percentage of total effect mediated by each of the mediators.

**TABLE 2:** Direct and indirect effects of land size on women's dietary diversity and body mass index

Mediator	Direct effect of land size on outcome (95% CI)	ACME (95% CI): Indirect effect of land size on outcome through mediator	% of total effect mediated (95% CI)
**Outcome: Women's dietary diversity**			
Production diversity score	0.039 (0.003, 0.073)	0.008 (0.003, 0.015)	17.6 (10.0, 60.4)
Value of agriculture production	0.044 (0.007, 0.080)	0.003 (−0.008, 0.013)	6.3 (3.6, 24.1)
Women's decision‐making	0.049 (0.012, 0.084)	−0.001 (−0.005, 0.001)	−3.0 (−10.9, −1.7)
Women's group participation	0.044 (0.008, 0.079)	−0.001 (−0.002, 0.002)	−1.2 (−5.6, −0.6)
Women's work‐free time	0.047 (0.011, 0.082)	−0.003 (−0.005, −0.001)	−6.2 (−27.1, −3.5)
Women's land ownership	0.039 (0.003, 0.743)	0.003 (−0.003, 0.009)	6.0 (3.1, 28.3)
**Outcome: Women's body mass index**			
Production diversity score	0.001 (−0.071, 0.070)	−0.031 (−0.048, −0.017)	64.6 (−104.8, 91.6)
Value of production	0.017 (−0.053, 0.083)	−0.047 (−0.075, −0.021)	96.3 (−205.8, 133.9)
Women's decision‐making	−0.018 (−0.089, 0.050)	−0.011 (−0.019, −0.004)	22.8 (−413, 393)
Women's group participation	−0.029 (−0.100, 0.039)	0.0003 (−0.002, 0.003)	−0.67 (−13.7, 11.5)
Women's work‐free time	−0.030 (−0.101, 0.039)	−0.005 (−0.010, −0.001)	10.5 (−146.1, 219.7)
Women's land ownership	−0.048 (−0.127, 0.028)	−0.0002 (−0.003, 0.003)	0.4 (−4.0, 5.1)

Abbreviations: ACME, average causal mediation effects; CI, confidence interval.

#### 3.3.1 Agricultural mediators

The results in Figure 2 show that household land size positively affects production diversity [*β* 0.215; 95% CI (0.171, 0.259)] and value of agricultural production [*β* 0.36; 95% CI (0.311, 0.409)]. There is evidence of a small positive effect of production diversity on women's dietary diversity [*β* 0.038; 95% CI (0.011, 0.065)], but there is no evidence of an effect of value of production on maternal dietary diversity. Consistent with this, the results in Table 2 show that production diversity partially mediates the positive effect of land size, with an indirect effect of 0.008 [95% CI (0.003, 0.015)] accounting for 17.6% of total effect mediated [95% CI (10.0, 60.4)].

#### 3.3.2 Women's empowerment mediators

Land size positively affects women's participation in decision‐making about agricultural processes [*β* 0.214; 95% CI (0.146, 0.283)], participation in community groups [*β* 0.079; 95% CI (0.015, 0.143)] and women's land ownership 0.124 [95% CI (0.011, 0.237)], but there is no evidence of an effect on women's work‐free time (Figure 2). There is no evidence of an effect of women's decision‐making on dietary diversity, but all three other hypothesised empowerment mediators (group participation, land ownership and work‐free time) positively affect maternal dietary diversity.

The results in Table 2 suggest that, despite there being no evidence of an effect of household land size on women's work‐free time, of the hypothesised women's empowerment mediators, only women's work‐free time partially mediates the association between land size and dietary diversity with an indirect effect of −0.003 [95% CI (−0.005, −0.001)] accounting for −6.2% (−27.1, −3.5) of the total effect. The negative coefficient of the indirect effect suggests that the positive effect of larger land size on dietary diversity is partially supressed by a woman having more work‐free time. This appears to be because, although there is a positive association between work‐free time and dietary diversity, an increase in land size leads to a reduction in work‐free time.

### 3.4 Pathways from land size to maternal BMI through agriculture and women's empowerment

**FIGURE 3:**
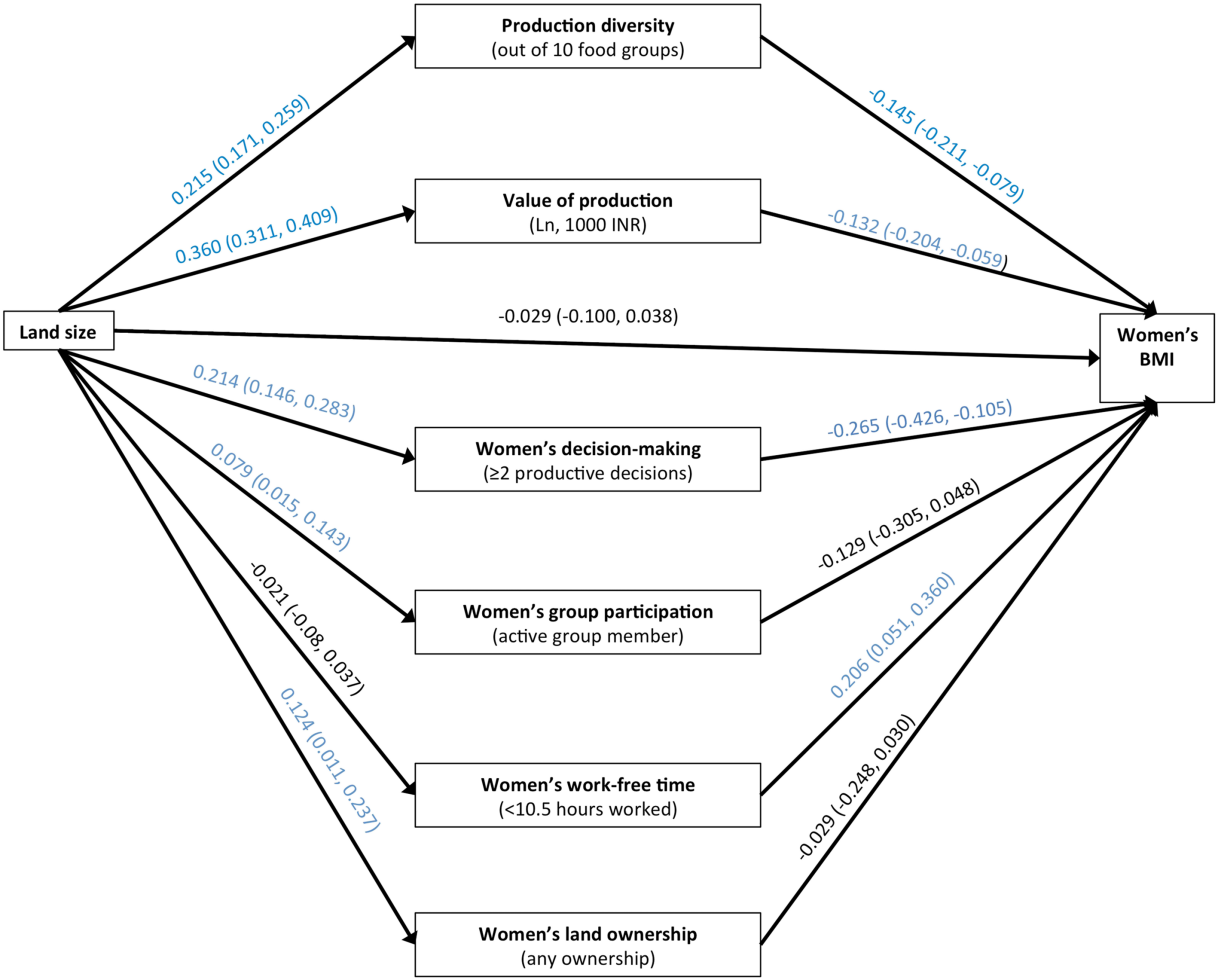
Pathways from land size to maternal body mass index (BMI) diet diversity: Estimations from potential outcomes framework analysis. All coefficients are from linear and logit regressions. All models adjust for caste group, years of maternal education, asset score, household size, female‐only households, maternal age and clustered study design. Estimations of associations between mediators and outcome are adjusted for exposure (ln land size)

Figure 3 gives the total effect of land size on maternal BMI along with the effects of land size on the hypothesised mediators and the effects of the mediators on maternal BMI (adjusted for land size). Again, consistent with Figure 1, there is no evidence of a total effect of household land size on maternal BMI [*β* −0.029; 95% CI (−0.100, 0.038)].

Table 2 gives the estimates of the direct effect of land size on maternal BMI and the indirect effects through each hypothesised mediator. Note the null total effect means that here, estimating the proportion mediated is problematic.

#### 3.4.1 Agricultural mediators

Household land size positively affects both production diversity and value of agricultural production. Additionally, both hypothesised agricultural mediators negatively affect maternal BMI. For both agricultural production mediators, we observe almost complete suppression of the effect of land size on maternal BMI, with no evidence of a direct effect for either mediator but indirect effects of −0.031 (−0.048, −0.017) through production diversity and −0.047 (−0.075, −0.021) through value of production. This appears to be because although there is a positive association between land size and the hypothesised mediators, production diversity and value of production, an increase in both mediators leads to a reduction in BMI, giving a null effect.

#### 3.4.2 Women's empowerment mediators

Household land size positively affects women's participation in decision‐making about agricultural processes, participation in community groups and women's land ownership but has minimal effect on women's work‐free time. There is evidence of a negative effect of women's decision‐making on maternal BMI [*β* −0.265; 95% CI (−0.426, −0.105)] and a positive effect of women's work‐free time on maternal BMI [*β* 0.206; 95% (0.051, 0.360)].

In Table 2, we see a similar pattern to that observed for the hypothesised agricultural mediators for women's decision‐making: an increase in land size positively affects women's decision‐making in agriculture, but an increase in decision‐making negatively affects maternal BMI. However, here the suppression is only partial with an indirect effect of −0.011 [95% CI (−0.019, −0.004)]. A different pattern is observed for women's work‐free time: with an increase in land size leading to a small decrease in work‐free time but an increase in work‐free time leading to an increase in BMI. The indirect effect of household land size on maternal BMI through work‐free time is −0.005 (−0.010, −0.001), suggesting that the positive effect of work‐free time on maternal BMI is suppressed by the negative effect of household land size on work‐free time.

Sensitivity analyses supported the results shown in Tables 2.

## 4 DISCUSSION

To our knowledge, this is the first study to unpack the pathways from land size to maternal nutrition. We find that the pathways between household land size and women's nutrition outcomes are complex, often acting in opposing directions. First, we find small effects between land size and dietary diversity but not maternal BMI. Second, agricultural production indicators appear to partially mediate these effects, by improving diets but compromising BMI. We see agriculture production mediators acting as ‘suppressors’ of the effect of land on BMI; that is, the total effect of land size on BMI (shown in Figure 3) is statistically not significant because the positive direct effects of land and the negative indirect effects of the mediator cancel each other out.

Third, different dimensions of women's empowerment are important independent determinants of maternal dietary diversity and BMI outcomes. Women's work‐free time partially mediates the effect of land size on dietary diversity and BMI. However, the positive effect of larger land size on dietary diversity is partially supressed by a reduction in work‐free time. Similarly, positive effect of work‐free time on maternal BMI is suppressed by the negative effect of household land size on work‐free time. Fourth*,* we find that an increase in land size leads to an increase in decision‐making but an increase in decision‐making leading to a decrease in maternal BMI.

The study was conducted in a rural Indian context, where around three quarters of the population earn their livelihood from agriculture (Odisha District Website, 2019), so we expected land to be an important asset for nutrition. However, the differing direct effects on dietary diversity (small and statistically significant) and BMI (small and statistically insignificant) are consistent with a review of other studies from South Asia (Shankar et al., 2019). The *Vasundhara* scheme in Odisha requires each homestead‐less household to be allocated 10 decimals (~0.01 acres) of homestead land, which may explain the very low percentage of households owning no land (Government of Odisha, 2018). However, these land distribution schemes have been criticised for being ineffective due to the low quality of land provided (Deo, 2011). Variable quality of land, along with other agricultural inputs (technology, labour and climatic factors such as rainfall) may explain some disconnect between land ownership and agricultural production (Rahman, 2010; Zepeda, 2001). Beyond subsistence agriculture, with increasing reliance on migration, wage labour including agricultural wage labour and non‐farm businesses, other non‐farm sources of income may be more important for improving nutrition outcomes (Shankar et al., 2019). Furthermore, diversifying agricultural production may require relatively little land. Chickens (which can produce both meat and egg food groups) can be kept near the homestead; vegetables can be grown on small kitchen gardens, and people may also collect fruits or other foods from publicly owned forest land.

The relatively weak linkages from agricultural production to dietary diversity are consistent with the growing body of evidence on this relationship (Dillon, McGee, & Oseni, 2015; Jones, Shrinivas, & Bezner‐Kerr, 2014; Sibhatu, Krishna, & Qaim, 2015). A disconnect may be explained by households selling their produce (Singh, Squire, & Strauss, 1986) or allocating the food to household members other than women (Harris‐Fry et al., 2018). The temporal mismatch in measurement may also explain a weak association, with production being measured over 1 year and diets over 1 day.

The negative association between agricultural production and maternal BMI suggests that improvements in diets may be insufficient to compensate for the women's energy expenditure required to participate in agriculture. Increasing agricultural production may require more physical activity, placing women in negative energy balance. The same could apply for women's decision‐making, if women who control decisions about agricultural production also take responsibility for carrying out those decisions. This hypothesis is corroborated by the finding that women with more work‐free time have higher BMIs (Figure 3).

Women's active participation in community groups—perhaps indicating social capital and extra‐household support—women's work‐free time and land ownership are all positively associated with dietary diversity, but they do not vary by household land size, suggesting that these indicators are independently important for maternal nutrition. Although these results contrast the null effects of women's group membership and workload on women's diets found by another study in Nepal (Malapit, Kadiyala, Quisumbing, Cunningham, & Tyagi, 2015), they agree with the overall conclusions that different domains of empowerment matter for diets. Consistent with the Nepali study, we find that women's work‐free time is positively associated with BMI. Our findings of negative effects of decision‐making are surprising, but other studies from Nepal and Bangladesh have also found null but negative direction of effect (Malapit, Kadiyala, et al., 2015; Sraboni, Malapit, Quisumbing, & Ahmed, 2014). It is possible that women who take more decisions also take more responsibility for the decisions made and therefore have heavier work burdens.

Our study benefits from a large sample size, a statistical counterfactual approach and detailed information on both agricultural production and nutrition outcomes. We note some important limitations. Measurement of land is challenging for many reasons: it relies on self‐reported sizes by people who may not know how much land they have (and may be unable to differentiate between land owned by the nuclear vs. extended family), may have difficultly estimating such sizes, may over‐report if larger land size is considered socially desirable or may under‐report in case it compromises their eligibility for entitlements or they have ‘encroached’ on government land. Research on estimation errors, and overreporting or underreporting of land size is needed to determine the possible implications for our findings. Furthermore, as noted earlier, none of these measures capture quality of land, which could plausibly be higher in better‐off households, perhaps leading to an underestimate of the effect of land on maternal nutrition. Another challenge is the differences in reference periods for annual agricultural production compared with shorter‐term dietary intakes. BMI does provide a longer‐term indicator of nutritional status, but we are unable to explore associations with dietary habits or seasonal changes. Repeated measurements over time would enable us to unpack seasonal changes in production and consumption patterns, workloads and the variance in the strength of associations between land use, production and food intakes at different points of year. It is worth noting that the CIs are also wide, and the results should be cautiously interpreted. Although low BMI indicates chronic energy deficiency and high BMI predicts future morbidity, BMI does not indicate fat distribution or body composition. Dietary Diversity Score is a food group diversity score and is a proxy for micronutrient adequacy. Whether or not intakes are adequate depends on dietary diversity as well as quantities of nutrient‐dense foods consumed, which our study does not measure.

Our research questions guiding the paper emerged after the definition of the study design and data collection. In particular, our research question arose due to concern of excluding the landless in our own (and other agricultural) interventions for which these data were collected. Thus, variable specifications, especially of mediators, were guided by the data available. Our application of counterfactuals to identify mediated effects using potential outcomes framework for causal mediation offers a rigorous analytical approach for a cross‐sectional study, especially given that that our analysis satisfies the first assumption of sequential ignorability. However, our study cannot account for all confounding, especially due to time‐varying unobservables. As such, any causal inference should be made cautiously. Future work using experimental or quasi‐experimental study designs is needed to further test these pathways.

Our study suggests that land transfer programmes may need to be coupled with other agricultural inputs, such as soil quality, fertiliser, irrigation and labour to improve nutrition status and should support more equitable workload allocation. This is consistent with other studies showing weak nutritional effects of land‐titling schemes in India (West Bengal; Santos, Fletschner, Savath, & Peterman, 2014), Vietnam (Menon, Van Der Meulen Rodgers, & Nguyen, 2014) and Ethiopia (Muchomba, 2017). This is especially the case where such land transfer programmes are for homesteads, where poor land quality might mean low potential for kitchen gardens. Nutrition‐sensitive agriculture interventions should consider reliance on land and land quality for food production because land size does seem to partially determine agricultural production and, in turn, nutritional outcomes. However, these effect sizes are small, and it is possible that, in the Odisha context where homestead‐less households have been provided with some land, agricultural diversification can be achieved on small plots. Other study contexts, where more people do not have any land, may require more careful programme design. The negative association between agricultural production and BMI indicates that programmes aiming to increase or diversify production would need to carefully consider their implications for women's and intra‐household allocation of labour (Johnston, Stevano, Malapit, Hull, & Kadiyala, 2018). This may partially explain the observed discrepancy between the larger, more consistently positive effects of nutrition‐sensitive agricultural interventions on dietary intakes compared with anthropometric outcomes (Ruel et al., 2018).

Further research is needed on the trade‐offs between improvements in household agricultural productive assets; women's empowerment in agriculture and women's nutritional outcomes; and how to mitigate these trade‐offs (women's work‐free time and energy expenditure) to optimise women's nutritional outcomes.

## CONFLICTS OF INTEREST

The authors declare that they have no conflicts of interest.

## CONTRIBUTIONS

SKa, EA and AP designed the trial. SG, SM, HH‐F and S. Kr contributed development of all the study tools, with inputs from all the co‐authors. SG, SM, HH‐F and SKa provided oversight for data collection. HH‐F and EA co‐led the data analysis for this paper with support from EB and oversight from SKa. HH‐F wrote the paper with support from SKa and EA. All authors provided critical feedback on earlier drafts and read and approved the final manuscript.

## Supporting information

Data S1. Supporting Information
