# Supplementary material for: Agricultural and empowerment pathways from land ownership to women's nutrition in India
Source: Matern Child Nutr. 2020 Mar 20;16(4):e12995. doi: 10.1111/mcn.12995 (PMC7507043; doi:10.1111/mcn.12995)
Supplement: Supplementary file 1 — Data S1. Supporting Information [file MCN-16-e12995-s001.docx]

## Pathways from land to women’s nutrition in rural India: Do agricultural production and women’s empowerment mediate the relationship between land size and women’s nutrition

Table 1 Variables used in analyses

| **Hypothesis** | **Indicator** | **Variable construction** |
| --- | --- | --- |
| Exposure | Land size | Self-reported size of landholdings owned by the household in natural log-transformed (Ln) acres. Land includes homestead land, agriculture land, and any other land. Households may have a Record of Rights, a share of ancestral land, or land with no record, including encroached land. |
| Nutrition outcomes | Women’s dietary diversity | Count out of ten food groups consumed by female caregivers aged 15-49 years, calculated using the Minimum Dietary Diversity for Women (MDD-W) (FAO, 2014). The ten food groups are: (1) grains, white roots and tubers, (2) pulses (beans, peas and lentils), (3) nuts and seeds, (4) dairy, (5) meat, poultry and fish, (6) eggs, (7) dark green leafy vegetables, (8) other vitamin A-rich fruits and vegetables, (9) other vegetables and (10) other fruits. |
|  | Women’s BMI | Body-mass index (kg/m^2^), in non-pregnant, non-postpartum women |
| Hypothesised agricultural mediators | Value of agricultural production | Ln- value of total agricultural production in the last 3 agricultural seasons (in the last 12 months), in 1000 Indian Rupees, calculated as: (quantity of each crop or livestock produced ×prices). Production from all cultivated land (owned, rented, shared or any other arrangement such cultivating on an extend family member’s land or community land) included. |
|  | Production Diversity | Count of 10 food groups produced, regardless of land ownership status, in the last 3 agricultural seasons ( in the last 12 months) by households in any quantity: (1) grains, white roots and tubers, (2) pulses (beans, peas and lentils), (3) nuts and seeds, (4) dairy, (5) meat, poultry and fish, (6) eggs, (7) dark green leafy vegetables, (8) other vitamin A-rich fruits and vegetables, (9) other vegetables and (10) other fruits (Berti, 2015). Production from all cultivated land (owned, rented, shared or other arrangements such cultivating on an extend family member’s land or community land) included. |
| Hypothesised women’s empowerment mediators* | Women’s decision-making | Women’s self-reported involvement in some or most/all in ≥ 2 vs < 2 productive decisions in the household, out of four possible decisions: food cropping, cash cropping, livestock, non-farm business decisions, based on the abbreviated Women’s Empowerment in Agriculture Index (H. Malapit et al., 2014). |
|  | Women’s group participation | Women’s self-reported active participation in any of the following community groups: farmer’s clubs, water and sanitation group, forest users’ groups, credit or microfinance institution, village development group, religious groups, Self-Help Groups, school-based groups, nutrition-related groups, or youth clubs. Based on the abbreviated Women’s Empowerment in Agriculture Index questions (“Is there a [group] in your community; Are you an active member of this group?”) (H. Malapit et al., 2014). |
|  | Women’s time use | Amount of work-free time that women have ( <10.5 vs ≥ 10.5 hours of work) based on a 24-hour time-use recall questionnaire, based on the abbreviated Women’s Empowerment in Agriculture Index (H. Malapit et al., 2014). |
|  | Women’s land ownership | Women’s self-reported land ownership, in two categories: none vs joint or sole ownership. |

*Below is a brief description of how we adapted abbreviated Women’s Empowerment in Agriculture Index (A-WEAI) indicators for women’s empowerment in our study and the rationale:

- **Input in productive decisions and income**: We combined the question on women’s decision-making over agricultural activities and women’s decision-making over income from the activities based on our pre-testing of these questions in our study site. We found that, in this context, these answers were difficult to disentangle, and results overlapped considerably. We also did not ask about decision-making about fishing, since the study setting is far inland, and fishponds are not common.
- **Ownership of assets**: Although we collected data on women’s ownership of multiple household assets, we selected women’s ownership of land as our mediator in this study. Rather than use the aggregated indicator of multiple assets, women’s ownership of land relates directly to our exposure of interest (total land size owned by the household).
- **Group membership**: We used this indicator as per the A-WEAI.
- **Women’s time use**: We used this indicator as per the A-WEAI
- **Credit**: We did not use the indicator on credit because, in our study context, credit is not common. In our sample, only 16.8% of households took any credit and, of those, around one third would not / were not sure that they would have taken the loan in hindsight. Credit is contentious in India and also may not be an appropriate indicator of ‘empowerment’.

*Sources:*

- - Supriya Garikipati, Isabelle Agier, Isabelle Guérin & Ariane Szafarz (2017) The Cost of Empowerment: Multiple Sources of Women’s Debt in Rural India, The Journal of Development Studies, 53:5, 700-722, DOI: [10.1080/00220388.2016.1205734](https://doi.org/10.1080/00220388.2016.1205734)
  - Banerjee, Abhijit, Esther Duflo, Rachel Glennerster, and Cynthia Kinnan. (2015). "The Miracle of Microfinance? Evidence from a Randomized Evaluation." *American Economic Journal: Applied Economics*, 7 (1): 22-53.
  - Supriya Garikipati (2008) The Impact of Lending to Women on Household Vulnerability and Women’s Empowerment: Evidence from India. World Development. V[olume 36, Issue 12](https://www.sciencedirect.com/science/journal/0305750X/36/12): 2620-2642
  - <https://www.marketwatch.com/press-release/over-indebtedness-and-its-drivers-among-microfinance-borrowers-in-india-2019-02-19>
  - <https://www.businessinsider.com/hundreds-of-suicides-in-india-linked-to-microfinance-organizations-2012-2?r=US&IR=T>
